# Comparison of *Mycobacterium avium* subsp. *paratuberculosis* infection in cattle, sheep and goats in the Khuzestan Province of Iran: Results of a preliminary survey

**DOI:** 10.1002/vms3.559

**Published:** 2021-07-06

**Authors:** Mahdi Pourmahdi Borujeni, Mohammad Rahim Haji Hajikolaei, Masoud Ghorbanpoor, Hamzeh Elhaei Sahar, Saeed Bagheri, Sanaz Roveyshedzadeh

**Affiliations:** ^1^ Department of Food Hygiene, Faculty of Veterinary Medicine Shahid Chamran University of Ahvaz Ahvaz Iran; ^2^ Department of Clinical Sciences, Faculty of Veterinary Medicine Shahid Chamran University of Ahvaz Ahvaz Iran; ^3^ Department of Pathobiology, Faculty of Veterinary Medicine Shahid Chamran University of Ahvaz Ahvaz Iran

**Keywords:** epidemiology, Johne's disease, *Mycobacterium avium* subsp. *paratuberculosis*, prevalence, serology

## Abstract

**Background:**

Paratuberculosis or Johne's disease, the chronic infectious granulomatous enteritis of ruminants, is a worldwide infection, which is caused by *Mycobacterium avium* subsp. *paratuberculosis* (MAP). The most common symptoms of this disease in cattle are loss of milk production, weight loss and diarrhoea, whereas in sheep and goats, the symptoms are emaciation, anorexia and severe disability.

**Objectives:**

The aim of this study was to compare the seroprevalence of MAP in cattle, sheep and goats in the southwest of Iran.

**Methods:**

Blood samples were randomly collected from 530 cattle, 568 sheep and 368 goats in southwest of Iran. Sera were tested by a commercial ELISA kit (ID vet; ID Screen® Paratuberculosis Indirect) for detection of antibodies of MAP.

**Results:**

Overall apparent and true seroprevalence rate of MAP was 6.00% (95% CI: 4.90%–7.30%) and 13.25% (95% CI: 11.55%– 14.95%). Apparent and true seroprevalence of MAP, respectively, was 4.34% (95% CI: 3.88%–6.46%) and 9.19% (95% CI: 6.98%–11.98%) in cattle, 6.87% (95% CI: 5.05%–9.27%) and 15.37% (95% CI: 12.60%–16.60%) in sheep and 7.07% (95% CI: 4.82%–10.18%) and 15.86% (95% CI: 12.41%–20.01%) in goats, respectively. As a result, there was no significant relationship between animal species and MAP infection. Moreover, multivariate logistic regression showed that the infection rate is not associated with age, gender and geographical location in cattle, sheep and goats (*P* > 0.05).

**Conclusion:**

This study confirms that the seroprevalence of MAP is relatively considerable in the cattle, sheep and goats in the southwest of Iran, although in cattle, it is less than goats and sheep. Therefore, preventive and control measures should be considered by animal health authorities and meat and dairy processing units.

## Graphical abstract

The present epidemiological study evaluated and compared the seroprevalence of MAP with the ELISA method in the cattle, sheep and goats following the detection of clinical cases. Moreover, the relationship between the infection and determinants such as species, age, gender and geographical location was investigated

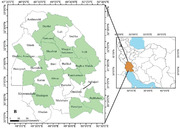

## 1 INTRODUCTION

Paratuberculosis or Johne's disease (JD), which is a chronic infectious granulomatous enteritis of ruminants, is caused by *Mycobacterium avium* subsp. *paratuberculosis* (MAP). Although it can be seen in cattle, sheep, goats and wild ruminants, it has been described more frequently in cattle (Manning, 2011; Sweeney et al., 2012; Whittington & Sergeant, 2001). Because of the economic importance of the JD to livestock around the world, it has been announced as one of the priorities of infection disease by the World Organization for Animal Health (OIE, 2020). Four stages of paratuberculosis in cattle have been described: silent infection, subclinical disease, clinical disease and advanced clinical disease. In the silent infection, there are no clinical signs and no effects on body weight (BW) gain or body condition. In the subclinical disease, carrier adults show no specific clinical signs but may be affected by other abnormalities such as mastitis or infertility. In the clinical disease, there is gradual loss of BW despite a normal appetite, and diarrhoea develops several weeks later. Milk production declines but vital signs are within normal limits. In advanced clinical disease, emaciation is the most obvious abnormality and is usually accompanied by intermandibular oedema, which has a tendency to disappear as diarrhoea develops. The course of the disease varies from weeks to months but always terminates in severe dehydration, emaciation and weakness with an ultimately fatal outcome (Constable et al., 2017). In sheep and goats, the most common clinical signs are emaciation, anorexia and severe disability (Attili et al., 2011; Constable et al., 2017). This disease is also important from the standpoint of public health because the cause of JD is likely related to Crohn's disease in human (Waddell et al., 2016). In the same vein, positive and consistent associations between MAP and Crohn's disease have been reported by Waddell et al. (2015) in a systematic review and meta‐analysis study. The main route of transmission of paratuberculosis is widely accepted to be through oral uptake of MAP by susceptible animals via ingestion of contaminated milk, water and other feed products or uptake from the environment (Constable et al., 2017; Manning, 2011; Sweeney et al., 2012). There are three groups of MAP strains that appear to correlate with the host of origin and are designated as ‘sheep‐type’ (type S), ‘cattle‐type’ (type C) and ‘bison‐type’ (type B). Type B strains are a subtype of type C and not restricted to Bison species (Bryant et al., 2016). However, the relationship between strain type and the host species is neither absolute nor always clear. For example, type S strains are more frequent in New Zealand beef cattle than type C strains where these species are frequently grazed together (Verdugo et al., 2014). Type C strains are isolated from a broad range of hosts and do not appear to have a host preference (Moloney & Whittington, 2008). The evidence for interspecies transmission is compelling, but the risk of natural transmission of type S strains from sheep and goats to cattle is low and occurs when susceptible animals are exposed to high doses of MAP only (Moloney & Whittington, 2008).

Due to the long incubation period of JD and the role of cattle, sheep and goats as incubatory carrier and distributor of the MAP with no signs, early identifying the infection is very important. In the same line, the best strategy to prevent the infection is to identify and cull the infected animals. Diagnosis of paratuberculosis is possible through molecular, culture and serology methods, which are less sensitive. The sensitivity of these methods may vary according to the characteristics of the cow, stage of infection and stage of lactation. For example, the sensitivity of the faecal culture varies with the stage of infection. In clinical cases, fecal culture sensitivities of 70% and higher were reported, whereas in clinically healthy but infected cows, the sensitivity of faecal cultures was reported to range between 23% and 29% in comparison with enzyme‐linked immunosorbent assay (ELISA) that was 7% and 39%. In general, the sensitivity and specificity of different methods of serology are reported as follows: complement fixation test 90% and 70%, agar gel immunodiffusion assay 96% and 94% and ELISA 45% and 99%, respectively (Constable et al., 2017). Thus, the infection rate is estimated less than the true value, and accordingly the iceberg phenomenon is seen in this disease (Magombedze et al., 2013; Nielsen & Toft, 2008; OIE, 2018). Moreover, the definite diagnosis is possible through time‐consuming culture; but the cheapest and fastest method is ELISA, which is a suitable diagnostic tool for detecting antibodies against this organism on a large scale (Ricchi et al., 2017; Tiwari et al., 2006). The ELISA response to MAP may also vary according to the characteristics of the cow and stage of lactation so that subclinical, light‐shedding cattle are usually seronegative, whereas heavily infected animals are usually seropositive. In most cows, in the early stages of infection when faecal shedding is low, the humoral antibody response is below the limit of detection, and currently available serologic tests are inadequate to detect those animals. As the infection progresses, the humoral response increases, and heavy faecal shedders and clinically affected animals are more readily detected (Constable et al., 2017).

Due to the economic importance of paratuberculosis, many studies have been conducted on its epidemiology throughout the world. In Iran also, the prevalence of this disease varies in cattle between 2% and 59% (Anzabi et al., 2005, 2009; Ghaemmaghami et al., 2012; Haji Hajikolaei et al., 2006; Heidarnejhad et al., 2017; Karimi et al., 2012; Khakpoor et al., 2012; Nassiri et al., 2012; Zarei et al., 2017a, 2017b) and in sheep and goats between 0.96% and 37% (Haji Hajikoulaei et al., 2002; Nemati, 2015). Despite the importance of the issue, no epidemiological study on seroprevalence and risk factors of MAP infection has been conducted in the Khuzestan Province of Iran. Therefore, the present study was aimed to identify both the infection rate of MAP in cattle, sheep and goats and the role of the risk factors including animal species, age, sex and geographical location. Knowing the prevalence rate of this chronic disease, the animal health authorities will be able to use the information to follow the prevention policies and provide the evaluation of control programs.

## 2 MATERIALS AND METHODS

### 2.1 Area of the study

**FIGURE 1:**
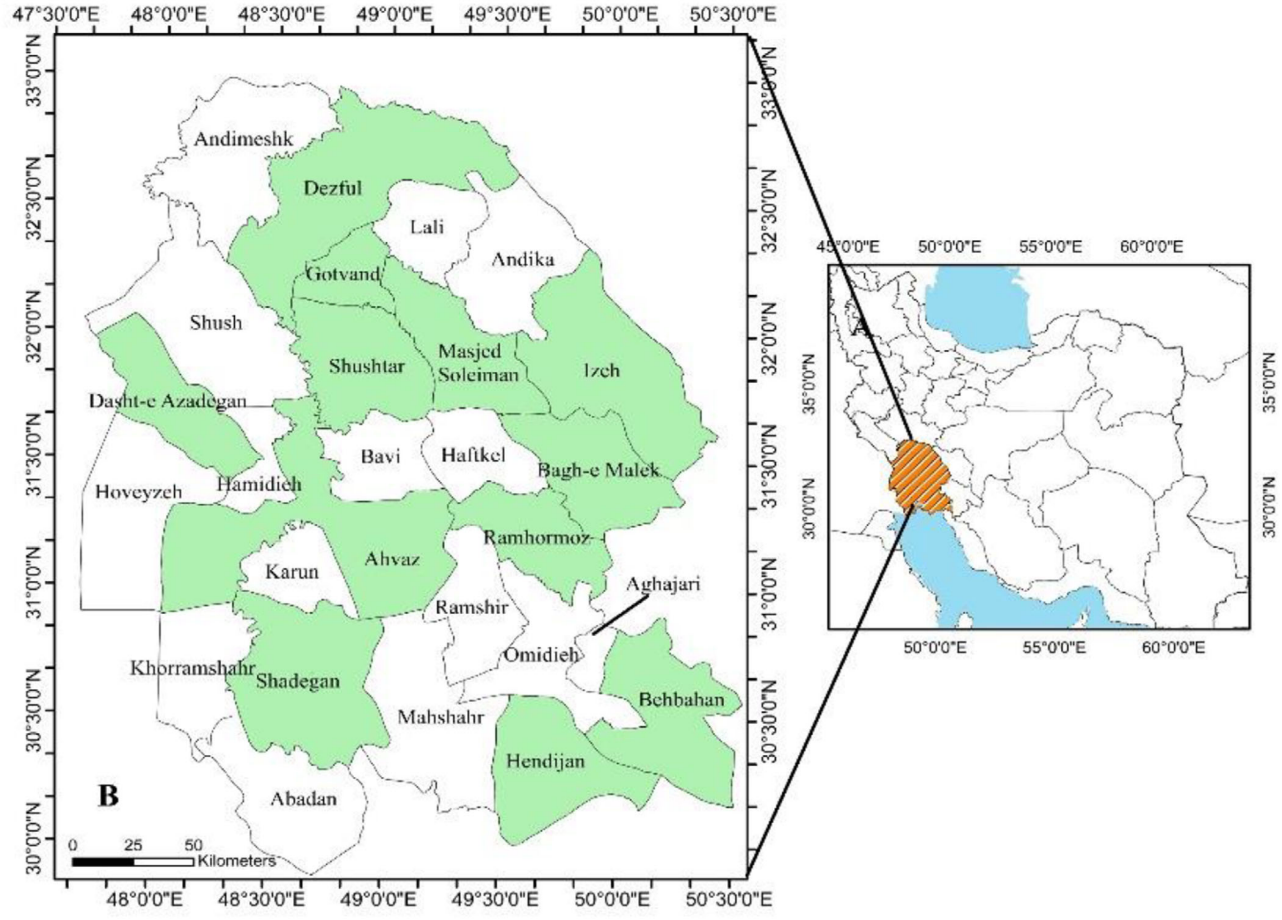
Location of sampled cities (green colour) in Khuzestan Province of Iran

The current cross‐sectional study was carried out in the Khuzestan Province located in the southwest of Iran (Figure 1). The topographic elevations of this tropical province, located between 48°E and 49.5°E longitudes and 31°N and 32°N latitudes, with an area of 63,213 km^2^ and 27 cities vary between 0 and 3740 m. The climate of this area varies from arid to humid. The northern parts of the province have cold weather, whereas the southern parts experience tropical weather (Zarasvandi et al., 2011). Therefore, to create regional differences in the epidemiological determinants such as environment and management, Khuzestan Province was divided into four different regions, out of which one or three cities were selected using simple randomisation. In Khuzestan Province, more than 300,000 cattle, 3.5 million sheep and 2.1 million goats, whose breeding is mostly traditional and somewhat semi‐industrial, are kept (Statistical Center of Iran, 2017).

### 2.2 Sample collection

For sampling from each farm, the individual animal was selected according to simple random sampling. Blood samples (10 ml) were taken from the jugular vein by venoject (EXPILAB, Gel & Clot Activator) from each animal. The required information on each sample, including animal species (cattle, sheep or goat), age (year), sex (male or female) and geographical location (Shushtar, Izeh, Dezful, Dasht‐ e Azadegan, Shadegan, Hendijan, Ramhormoz, Bagh‐ e Malek, Behbahan, Masjed Soleyman, Gotvand or Ahvaz) were documented. The mean and standard deviation of the age of cattle were 4.12 ± 2.66, sheep 3.98 ± 1.59 and goats 3.17 ± 1.73 years. The selected animals were also divided into three age groups (≤2, 2–4 and ≥5 years old). Because of the pathogenesis of the MAP, the long incubation period, seroconversion is unlikely in the young animals. So all of the examined animals were older than 6 months. The absolute frequency of samples based on independent variables is summarised in Tables 1, 2 and 3.

**TABLE 1:** Seroprevalence of *Mycobacterium avium* subsp. *paratuberculosis* (MAP) infection in cattle in southwest of Iran based on age, sex and geographical location

**Category**	**Groups**	**Apparent prevalence (AP; positive *N*/total *N*)**	**True prevalence (TP)**	**Odds ratio (OR)**	**95% CI for OR**	***P*‐value**
Age	≤2^a^	3.26% (3/92)	6.55%	1	–	–
	3‐4^a^	4.31% (9/209)	9.12%	1.34	0.35–5.05	0.66
	≥5^a^	4.80% (11/229)	10.31%	1.50	0.41–5.49	0.54
Sex	Male^a^	2.38% (1/42)	4.40%	1	–	–
	Female^a^	4.51% (22/488)	9.60%	1.94	0.25–14.73	0.52
Geographical location	Ramhormoz^b^	0% (0/59)	0%	–	–	–
	Dasht‐e Azadegan^b^	0% (0/60)	0%	–	–	–
	Dezful^ab^	1.92% (1/52)	3.27%	1	–	–
	Bagh‐e Malek^ab^	2.70% (2/74)	5.18%	1.42	0.13–16.05	0.78
	Shadegan^ab^	3.45% (2/58)	7.01%	1.82	0.16–20.69	0.63
	Ahvaz^ab^	4.44% (2/45)	9.34%	2.37	0.21–27.07	0.49
	Shushtar^ab^	6.98% (3/43)	15.64%	3.83	0.38–38.18	0.25
	Behbahan^ab^	7.41% (6/81)	16.69%	4.08	0.48–34.91	0.20
	Hendijan^a^	12.07% (7/58)	28.08%	7.00	0.83–58.96	0.07

*Note*: The different lowercase letters represent a significant difference.

**TABLE 2:** Seroprevalence of MAP infection in sheep in southwest of Iran based on age, sex and geographical location

**Category**	**Groups**	**AP (positive *N*/total *N*)**	**TP**	**Odds ratio**	**95% CI for OR**	***P*‐value**
Age	≤2^a^	6.06% (6/99)	13.39%	1.34	0.38–5.08	0.62
	3‐4^a^	7.65% (29/379)	17.28%	1.78	0.61–5.20	0.29
	≥5^a^	4.44% (4/90)	9.43%	1	–	–
Sex	Male^a^	2.22% (1/45)	4.01%	1	–	–
	Female^a^	7.27% (38/523)	16.35%	3.45	0.46–25.71	0.33
Geographical location	Bagh‐ e Malek^a^	3.70% (3/81)	7.62%	1	–	–
	Ahvaz^a^	4.42% (5/113)	9.38%	1.20	0.28–5.19	1
	Dasht‐e Azadegan^a^	5.00% (3/60)	10.80%	1.37	0.27–7.03	1
	Hendijan^a^	6.52% (6/92)	14.52%	1.81	0.44–7.50	0.62
	Dezful^a^	7.69% (5/65)	17.38%	2.17	0.50–9.43	0.49
	Masjed Soleyman^a^	10.71% (9/84)	24.76%	3.12	0.81–11.97	0.15
	Gotvand^a^	10.96% (8/73)	25.37%	3.20	0.82–12.56	0.15

*Note*: The different lowercase letters represent a significant difference.

**TABLE 3:** Seroprevalence of MAP infection in goats in southwest of Iran based on age, sex and geographical location

**Category**	**Groups**	**AP (positive *N*/total *N*)**	**TP**	**Odds ratio**	**95% CI for OR**	***P*‐value**
Age	≤2^a^	6.30% (8/127)	13.99%	1	–	–
	3‐4^a^	7.43% (13/175)	16.74%	1.19	0.48–2.97	0.70
	≥5^a^	7.58% (5/66)	17.11%	1.22	0.38–3.89	0.74
Sex	Male^a^	6.15% (4/65)	13.62%	1	–	–
	Female^a^	7.26% (22/303)	16.32%	1.19	0.40–3.59	0.96
Geographical location	Hendijan^a^	2.67% (2/75)	5.11%	1	–	–
	Izeh^a^	4.92% (3/61)	10.61%	1.89	0.31–11.68	0.81
	Dezful^a^	5.00% (3/60)	10.80%	1.92	0.31–11.89	0.80
	Ahvaz^a^	8.06% (5/62)	18.28%	3.20	0.60–17.11	0.30
	Dasht‐e Azadegan^a^	10.00% (5/50)	23.02%	4.06	0.76–21.79	0.18
	Shushtar^a^	13.33% (8/60)	31.16%	5.62	1.15–27.53	0.04

*Note*: The different lowercase letters represent a significant difference.

### 2.3 Serological analysis

The blood samples from a total of 1466 animals including 530 cattle, 568 sheep and 368 goats were transferred to the laboratory and centrifuged at 1000 × g for 10 min. Then, serum was slowly removed from the outer layer of the tube and was transferred to a coded microtube. Thereafter, microtubes were kept at –20°C until the checking time by a commercial indirect ELISA kit (ID vet; ID Screen® Paratuberculosis Indirect) for anti‐MAP antibodies. All the samples were tested according to the instructions of the company. Optical density (OD) of individual samples and positive (PC) and negative (NC) controls was read by an ELISA reader (Accua reader) at 450 nm. Then, based on the S/P percentage, the results were interpreted.

(1) $$S/P=\frac{OD_{Sample}-OD_{NC}}{OD_{PC}-OD_{NC}}\times 100.$$

According to the instructions of the kit, the samples with 70% S/P or more were considered positive, whereas those with S/P higher than 60% and lower than 70% were doubtful. Finally, samples with 60% S/P or lower were estimated negative.

### 2.4 Statistical analysis

Statistical analysis of the data was performed using SPSS (Version 16.0; SPSS Inc.). The association between animal species, age, sex and geographic location with infection was analysed by the Chi‐square test. In order to calculate the strength of association, univariate logistic regression was performed for each potential risk factor. Furthermore, in order to investigate the simultaneous effect of multiple factors under investigation and control of confounders on infection, a multivariate logistic regression model in a backward, stepwise algorithm was used. The goodness of fit of the model was determined using the Hosmer and Lemeshow test. Moreover, the Mann–Whitney U and Kruskal–Wallis tests were used to compare the age of infected and non‐infected animals in one species and among species, respectively. Cramer's V coefficient value was calculated to determine the correlation between species and infection. Besides, the percentage of true prevalence (TP, the proportion of truly infected animals with MAP) was calculated based on apparent prevalence (AP) percentage (the proportion of positive animals in ELISA) and sensitivity (Se = 41.5%) and specificity (Sp = 99.4%) of ELISA kit (Fry et al., 2008) using the formula TP = (AP + Sp – 1)/(Se + Sp – 1). Also, the estimation of confidence intervals (CI) for proportion was calculated by the Agresti–Coull method (Thrusfield et al., 2018). Also, differences were considered statistically significant (*P* ≤ 0.05). The map was drawn using ArcGIS software version 10.3.

## 3 RESULTS

### 3.1 Seroprevalence of MAP

The overall apparent and true seroprevalence rate of MAP regardless to animal species was 6.00% (95% CI: 4.90%–7.30%) and 13.25% (95% CI: 11.55%–14.95%). Apparent and true seroprevalence rate of MAP, respectively, was 4.34% (23 cattle out of 530 cattle, 95% CI: 3.88%–6.46%) and 9.19% (95% CI: 6.98%–11.98%) in cattle, 6.87% (39 sheep out of 568 sheep, 95% CI: 5.05%–9.27%) and 15.37% (95% CI: 12.60%–16.60%) in sheep and 7.07% (26 goats out of 368 goats, 95% CI: 4.82%–10.18%) and 15.86% (95% CI: 12.41%–20.01%) in goats, respectively.

### 3.2 Association between animal species and MAP

There was no significant relationship between species and infection (χ^2 ^= 4.09, df = 2, *P *= 0.13). In comparison with the cattle, the odds of infection in the sheep and goats were 1.63 (95% CI: 0.96–2.76, *P *= 0.07) and 1.68 (95% CI: 0.94–2.99, *P *= 0.08), respectively; in this regard, 0.8% of fluctuation in infection was justified by the species.

### 3.3 Association between animal age and MAP

The Chi‐square test showed that the infection was not associated with age in cattle (χ^2 ^= 0.38, df = 2, *P *= 0.83), sheep (χ^2 ^= 1.29, df = 2, *P *= 0.52) and goats (χ^2 ^= 0.18, df = 2, *P *= 0.92); however, it increases with ageing in cattle and goats and decreases in sheep. The odds of infection between the age, based on the year, and disease in cattle and goats are 1.02 and 1.08, respectively, implying that the odds of infection increased 2% and 8% with rising 1 year of age. Moreover, 0.4% and 0.3% of fluctuation in infection were justified by age in cattle and goats, respectively. In sheep, the odds of infection between the age, based on the year, and disease is 0.96 implying that the odds of infection decreased 4% with rising 1 year of age. Furthermore, 0.1% of fluctuation in infection was justified by age (Tables 1, 2 and 3). The average age of infected cattle, sheep and goats was 4.2, 3.8 and 3.5 years, respectively, whose difference was not statistically significant (χ^2 ^= 2.9, df = 2, *P *= 0.24). Besides, the difference between the average age of infected/non‐infected cattle (4.2/4.1 years, *P *= 0.48), sheep (3.7/4 years, P = 0.3) and goats (3.5/3.2 years, *P *= 0.37) was not statistically significant.

### 3.4 Association between animal sex and MAP

Although the prevalence of MAP infection in female cattle, sheep and goats was higher than in males, there was no significant difference between these sex groups according to the Chi‐square test (in cattle: χ^2 ^= 0.42, df = 1, *P *= 0.52; in sheep: χ^2 ^= 0.95, df = 1, *P *= 0.33; in goats: χ^2 ^= 0.00, df = 1, *P *= 0.96). Moreover, the odds of infection in female cattle, sheep and goats were 1.94, 3.45 and 1.19, respectively, compared to those of males. Furthermore, 0.3%, 1% and 0.1% of fluctuation in infection were justified by gender in cattle, sheep and goats, respectively (Tables 1, 2 and 3).

### 3.5 Association between geographical location and MAP

The infection rates varied among different cities ranging from 0% to 12.07% in cattle (χ^2 ^= 17.62, df = 8, *P *= 0.02). However, the Chi‐square test showed that the infection was not associated with geographical location in sheep (χ^2 ^= 5.59, df = 6, *P *= 0.36) and goats (χ^2 ^= 7.37, df = 5, *P *= 0.2). Moreover, 12.3%, 2.9% and 4.9% of fluctuation in infection were justified by geographical location, respectively, in cattle, sheep and goats (Tables 1, 2 and 3). Cramer's V coefficient between infection rate in cattle, sheep and goats, in Ahvaz, Dezful, Hendijan and Dasht‐ e Azadegan, was 0.19, 0.07, 0.11 and 0.15, respectively.

### 3.6 Multivariate analysis

Multivariate logistic regression in cattle, sheep and goats showed that 12.4%, 4% and 5.3% of fluctuation on an infection with MAP were justified by age, sex and geographical location, respectively. However, in backward stepwise logistic regression, none of them had a significant effect on infection.

## 4 DISCUSSION

The present epidemiological study evaluated and compared the seroprevalence of MAP with the ELISA method in the cattle, sheep and goats without clinical signs of paratuberculosis in the southwest of Iran. Moreover, the relationship between the infection and determinants such as species, age, gender and geographical location was investigated. In MAP infection, there is a relationship between active infection and shedding with high serum antibody titre (Begg et al., 2018; Collins et al., 2005; Steuer et al., 2019). Collins et al. (2005) showed that there is a direct relationship between the magnitude of ELISA results and the odds of a cow shedding MAP. Despite its low sensitivity of ELISA (about 50%), a commercial indirect ELISA was selected for this study because of the convenience of sample collection and rapid laboratory procedure. Furthermore, this ELISA kit is used in Iran and displayed the highest overall accuracy (specificity of 99.42% and sensitivity of 41.5%) of four commercial ELISA kits investigated by receiver operating characteristic analysis in a previous study (Fry et al., 2008).

The results indicated that the apparent and true seroprevalence of MAP, respectively, is 4.34% and 9.19% in cattle, 6.87% and 15.37% in sheep and 7.07% and 15.86% in goats. In comparison with this study, the frequency of MAP infection in slaughtered cattle in Ahvaz abattoir was reported to be 3% by ELISA and 2% by Ziehl–Neelsen staining methods and 1.4% and 0.96% by Ziehl–Neelsen staining in sheep and goats, respectively (Haji Hajikoulaei et al., 2002, 2006; Zarei et al., 2017a, 2017b). Also, the MAP infection rate in cattle, in other areas of Iran with different environmental conditions, was reported to be 3.6% to 25% by ELISA, polymerase chain reaction (PCR), culture and Ziehl‐Neelsen staining methods (Anzabi et al., 2005; Anzabi et al., 2009; Ghaemmaghami et al., 2012; Heidarnejhad et al., 2017; Karimi et al., 2012; Khakpoor et al., 2012; Nassiri et al., 2012). The prevalence of MAP infection in cattle, in other countries, has been reported to be 2.31% to 70.4% (Botsaris et al., 2010; Chiodini & van Kruiningen, 1986; Collins et al., 1994; Gurung et al., 2018; Kaur et al., 2011; Lombard et al., 2013; Pillars et al., 2009; Pradhan et al., 2011; Verdugo et al., 2018; Vilar et al., 2015). The MAP infection rate in goats, in some area of Iran, was 37% and 17.3% by PCR and culture methods, respectively (Nemati, 2015), but it was 0.3% to 45.1% in other countries (Dimarelli‐Malli et al., 2009; Dixit et al., 2013; Kumthekar et al., 2013; Lee et al., 2006; Liapi et al., 2011; Martinez Herrera et al., 2012; Mpenda & Buza, 2014; Pithua & Kollias, 2012; Rerkyusuke et al., 2018; Stau et al., 2012; Villari et al., 2009). The prevalence of infection in sheep in some countries has been reported as 2.4% to 21.1% (Dimarelli‐Malli et al., 2009; Khamassi Khbou et al., 2020; Liapi et al., 2011; Morales‐Pablos et al., 2020; Sergeant & Baldock, 2002; Stau et al., 2012; Villari et al., 2009). This may be due to the difference in the sample size, sampling method, methods of examination, herd size and management, environmental and host determinants (Constable et al., 2017; Thrusfield et al., 2018).

In this study, the rate of infection in cattle, sheep and goats was similar, so the species is not a risk factor for it. Also, Cramer's V coefficient showed that there is a very slight relationship in seropositivity rates between these species in four cities, including Dasht‐e Azadegan, Ahvaz, Dezful and Hendijan. As explained earlier, this study was merely a serological study that cannot exactly determine the interspecies transmission of MAP in this area. Due to the fact that the relationship between strain and host is not clear, and between the two major strains of MAP, type C was isolated from a broad range of animals, and thus did not have a particular host; also type S mostly infect sheep and goats and therefore transmission from these species to cattle is low (Moloney & Whittington, 2008; Verdugo et al., 2014). So it cannot be concluded which type of MAP, C or S is the main type in this area. To answer this question, more studies especially from a molecular standpoint are needed.

In the present study, the age of all the three examined species was more than 6 months old and there was no significant relationship between age and infection in cattle, sheep and goats. Stau et al. (2012) and Morales‐Pablos et al. (2020) also proved that there was no relationship between age and infection in sheep and goats. However, Attili et al. (2011), Cetinkaya et al. (1996), Fecteau et al. (2010), Rerkyusuke et al. (2018), Karimi et al. (2012), Weber et al. (2010) and Woodbine et al. (2009) showed that age was significantly related to infection. Although according to the ELISA results, we are not able to determine the time of infection, because of no relationship between age and infection and the other hands, the best time for infection is the first month of life of the animals, it is concluded that the examined animals may be infected in the early stage of their life. Experimental and field studies showed that infection becomes more difficult when calves are 4 months or older, and susceptibility to infection from 1 year of age appears to be similar to that of adult animals (Constable et al., 2017). Therefore, the resistance to disease increases with age, so older animals appear susceptible to infection but relatively resistant to progression to disease (Marquetoux et al., 2018).

In this study, the relative frequency of positive cases in both females and males was the same. Also, Anderson et al. (1992), Constable et al. (2017), Karimi et al. (2012), Kimberling (1988), Rerkyusuke et al. (2018), Morales‐Pablos et al. (2020) and Stau et al. (2012) showed that there is no statistically significant relationship between infection and gender. Generally, the MAP, not attending to a specific gender, infects both males and females, and infection is not related to sex determinants such as hormonal, occupational, behavioural and genetic determinants (Thrusfield et al., 2018).

The effect of geographical location on MAP infection rate might be due to the difference in animal management such as herd size, health, feeding and stress. In this study, the relationship between geographical location and infection in cattle, sheep and goats was not statistically significant. In line with the results of the present study, Cetinkaya et al. (1996), Lombard et al. (2006) and Morales‐Pablos et al. (2020) indicated that there is no significant relationship between geographical location and infection. However, Singh et al. (2014) proved that there is a significant relationship between the above‐mentioned variables.

As a limitation of this study, we used a commercial ELISA kit; however, it has been shown that the direct faecal PCR method is more sensitive than ELISA in detecting animals potentially infected with MAP (Clark et al., 2008). In diagnosing the infection in young and newly infected animals, because of the lack of enough antibody production, the ELISA has less sensitivity; thus, the diagnosis power of ELISA increases with the advancement of the disease and the increase of antibody production (Sweeney et al., 1995). In this regard, Juste et al. (2005) examined the power of both ELISA and blood PCR in detecting MAP in cattle and sheep. They showed that each method would detect different stages of MAP infection because their respective targets (bacteria and antibodies) might not have parallel dynamics. The young animals were more easily diagnosed by PCR than by ELISA, possibly because of the rapid recirculation of MAP‐loaded phagocytic cells from the intestinal lymphoid tissue into other lymphoid tissues after the infection, reinfection or reactivation. This should be expected to be more frequent among young animals newly exposed to MAP than in adults known to be more resistant to infection. In contrast, because the antibody response is slow to develop and highly dependent on the total number of mycobacteria, the most advanced cases should have detectable antibody responses (Juste et al., 1994; van der Giessen et al., 1995).

## 5 CONCLUSION

The current study was merely a serological survey. Although the results of this study may be different in comparison to the other studies (less than some and more than others), the TP of 13.25% regardless of species of these examined animals, should be considered. Because the studied animals are kept mostly together, it is not possible to give a definite opinion about the interspecies transmission of MAP in these animals. Therefore, a molecular study or culture of faeces needs to determine this purpose. On the other hand, the prevalence rate of MAP in this area should be considered by the animal health authorities to prevent economic losses. So control procedures such as vaccination, keeping the newborn away from the infected mother and omitting and limiting the infected animals should be seriously followed. In addition, the function of meat and dairy processing units with applying good hygienic practice and pasteurisation could be effective to prevent human contamination from MAP in the southwest of Iran.

## AUTHOR CONTRIBUTIONS

Mahdi Pourmahdi Borujeni and Mohammad Rahim Haji Hajikolaei designed the work and wrote the manuscript. Mahdi Pourmahdi Borujeni, Mohammad Rahim Haji Hajikolaei, Masoud Ghorbanpoor, Hamzeh Elhaei Sahar, Saeed Bagheri and Sanaz Roveyshedzadeh performed experiments and acquired data. Mahdi Pourmahdi Borujeni analysed and interpreted the data. All authors read and approved the final manuscript.

### PEER REVIEW

The peer review history for this article is available at https://publons.com/publon/10.1002/vms3.559.

## ETHICS STATEMENT

This study was an ‘observational study’, and the research protocol was reviewed and approved by the research committee of the Faculty of Veterinary Medicine, Shahid Chamran University of Ahvaz and documented by the number: 97581113.

## CONFLICT OF INTEREST

The authors of this manuscript declare no conflict of interests.

## Data Availability

The data that support the findings of this study are available from the corresponding author upon reasonable request.
